# Atomic force microscopy characterization of kinase-mediated phosphorylation of a peptide monolayer

**DOI:** 10.1038/srep36793

**Published:** 2016-11-14

**Authors:** Roman Zhuravel, Einav Amit, Shir Elbaz, Dvir Rotem, Yu-Ju Chen, Assaf Friedler, Shlomo Yitzchaik, Danny Porath

**Affiliations:** 1 Institute of Chemistry and the Center for Nanoscience and Nanotechnology, The Hebrew University of Jerusalem, Safra Campus, Givat Ram, Jerusalem 91904, Israel; 2 Institute of Chemistry, Academia Sinica, Taipei, Taiwan

## Abstract

We describe the detailed microscopic changes in a peptide monolayer following kinase-mediated phosphorylation. A reversible electrochemical transformation was observed using square wave voltammetry (SWV) in the reversible cycle of peptide phosphorylation by ERK2 followed by dephosphorylation by alkaline phosphatase. A newly developed method for analyzing local roughness, measured by atomic force microscope (AFM), showed a bimodal distribution. This may indicate either a hole-formation mechanism and/or regions on the surface in which the peptide changed its conformation upon phosphorylation, resulting in increased roughness and current. Our results provide the mechanistic basis for developing biosensors for detecting kinase-mediated phosphorylation in disease.

Kinases play a central role in cellular signalling^1^. Phosphorylation mediated by abnormal kinase activity is strongly linked to cancer and therefore presents a promising target for its therapy^2^,^3^,^4^,^5^,^6^,^7^,^8^. Due to the medical and biological significance of kinases, many methods for detecting their activity were demonstrated. These include radiometric assays, spectroscopic assays and electrochemical methods^9^,^10^,^11^,^12^,^13^,^14^. Electrochemical characterization of kinase activity has the advantages of avoiding radioactive materials, high detection sensitivities and consumption of lower amounts of analyte without the need for amplification. In some electrochemical methods, the detection is based on measuring changes in electrochemical impedance caused by phosphorylation of dense peptide monolayer by a specific kinase. This is performed using electrochemical impedance spectroscopy (EIS)^15^.

To understand the microscopic changes in the monolayer and the mechanism of how the phosphorylation affects the monolayer, we developed a new analysis method and used it for characterizing the topography of a peptide monolayer. The monolayer was composed of the Hepatoma Derived Growth Factor (HDGF) 160–174 peptide, derived from the HDGF protein that is strongly related to several types of cancer^16^,^17^,^18^. Serine 165 on HDGF has elevated phosphorylation level in lung cancer and is likely to be phosphorylated by the ERK2 kinase^15^. We have already shown that phosphorylation of this peptide by ERK2 leads to decrease in impedance while its dephosphorylation by alkaline phosphatase (AP) increases the impedance^15^. Surface roughness changes in this peptide monolayer upon phosphorylation were previously measured by us on annealed gold substrates. The RMS roughness was measured by atomic force microscope (AFM) at atomically smooth terraces on the annealed gold before and after phosphorylation. After phosphorylation, the overall surface roughness was increased. Phosphorylation also widened the roughness distribution, indicating a less homogeneous surface^15^.

Here we studied the phosphorylation and dephosphorylation of the peptide monolayer using square wave voltammetry (SWV) and local roughness measurements with AFM. Previously we have shown that the EIS can be used as a sensitive method for characterizing the capacitance vs. resistance changes in the peptide monolayer^19^. SWV and EIS are both non-destructive, but SWV is a faster and more reproducible method. In spite of its simplicity it has great sensitivity for the detection of kinase-mediated phosphorylation, without the need of chemical labelling of the substrate or enzyme. The method involves the creation of a unique peptide monolayer on gold electrodes, and measurements of SWV before and after the enzymatic phosphorylation reaction.

Surface roughness is commonly calculated by averaging over the whole scanned area to provide a single value of the global rms roughness^20^,^21^,^22^. This straightforward approach is strongly affected by aggregates and contaminants that always exist on the surface in such experiments. It is also blind to delicate changes between different areas on the sample, e.g. it cannot observe if there are different typical roughness values at different parts of the sample.

To overcome these disadvantages, we divided the scanned areas to many smaller sub-areas and created an overall distribution of the rms roughness of each one of them. This method provides a much better sensitivity and enables to observe and distinguish between few roughness values on the same surface. It is also much less sensitive to large contaminants, such as aggregates of peptides, impurities in the solution including residues from preparation processes and atmospheric particles of various types and sizes that adsorb to the surface. With the current approach, these do not affect the distribution as they do in global rms roughness analysis, where they up-shift and widen the overall roughness distribution. Here, we measured the roughness changes of the peptide monolayer on ultra-flat gold surface^23^ upon kinase phosphorylation and phosphatase dephosphorylation reactions. The newly developed analysis method used here provides a deeper insight into the local microscopic changes on the surface.

## Methods

### Sample preparation

Ultra-flat gold substrates were prepared as follows^23^: 150 nm of gold were evaporated on freshly cleaved mica. A 5 × 5 mm^2^ glass was attached using epoxy glue (epotek 301-2, Epoxy Technology Inc.) and dried for 3 hours at 80 °C, then the mica sheet was removed by submerging in pure tetrahydrofuran (THF) for 3 minutes.

The surface was submerged overnight in a peptide containing solution (0.1 mM HDGF 160–174, with the sequence CDLLEDSPKRPKEAEN in 100 mM phosphate buffer, pH = 8) immediately after mica removal. Phosphorylation with GST – tagged ERK2 (New England Biolabs) was done by immersing the slides in 300 μl kinase reaction medium (3 μM kinase, 80 mM Tris-HCl pH = 7.5, 40 mM MgCl_2_, 0.2 mg/mL BSA and 100 μM ATP) at 37 °C. The reaction was initiated by adding freshly prepared protein kinase solution and stopped by rinsing, after 30 min.

For dephosphorylation, AP, 0.1 Units in a final reaction volume of 300 μl, was added for 30 minutes at 37 °C. 1 Unit AP was dissolved in a 1 ml of Tris borate buffer, (pH 8.5), containing 10 mM MgCl_2_. (One unit (U) AP is defined as the amount of enzyme that can hydrolyze 1 μmol of phosphorylated peptide in a total reaction volume of 1 ml in 1 min at 37 °C). After each step the surface was thoroughly washed in tri-distilled water and dried in N_2_ flow.

### Electrochemical Characterizations

Square Wave Voltammetry (SWV) measurements were performed using an Autolab PGSTAT12 digital potentiostat (EcoChemie BV, Utrecht, The Netherlands) connected to NOVA software. A conventional three-electrode cell was employed; a Platinum electrode as a counter electrode, a 2 mm gold electrode (CH instruments) as a working electrode, and a standard Ag/AgCl reference electrode (Metrohm) with a KCl concentration of 3.0 M. Peptide immobilization on the gold electrode was achieved by chemisorption via cysteine thiol functional-group anchoring to the Au surface. The peptide monolayer formation, phosphorylation by ERK2 and dephosphorylation by AP on the working electrode were performed in the same conditions and with the same solutions as on the gold surfaces (see above). The volume of the enzyme solutions used on each electrode was 40 μl. The electrodes were characterized by SWV after each modification step. The voltammograms were obtained by scanning from −0.3 to −0.6 V with a step potential of 25 mV, amplitude of 10 mV and frequency 25 Hz at a scan rate of 625 mV/s. To provide redox species, a 1 mM [Fe(CN)_6_]^3−^ solution additionally containing 100 mM KNO_3_ as supporting electrolyte was used. In the current study the distance between the working and counter electrodes was invariable.

### Roughness analysis

Topographic characterization was done in non-contact AFM (Smart-AIST, inc.) with soft cantilevers (OMCL-RC800PSA, Olympus inc. – Silicon Nitride tip with typical curvature of 15 nm, cantilever spring constant 0.76 N/m with typical resonance frequency of 71 kHz) to minimize tip impact on the surface. Initial image processing was carried out with WSxM 5.0 develop 7.0. Global roughness was calculated using WSxM on the whole image^24^.

**Figure 1:**
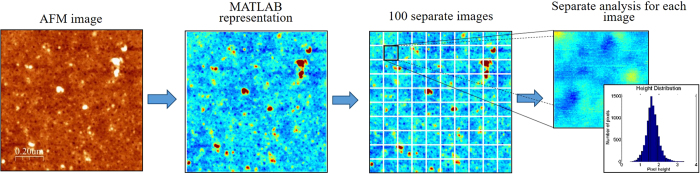
Local roughness analysis scheme. From left to right –AFM image acquisition of areas of 1×1 mm^2^ and initial image processing (plane subtraction and line removal), transfer the image to MATLAB, divide it to smaller images (100) and analyze each one separately.

The roughness of each scanned area was analysed in MATLAB as a matrix of 1000 × 1000 elements, each element representing the height of one pixel. i.e., 1 × 1 nm^2^. The matrix is divided to 100 small sub-matrices, 10,000 pixels each. RMS roughness is calculated for every sub-matrix of 100 × 100 pixels, as shown in Fig. 1. We define RMS roughness as follows:


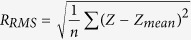


here Z is the height value measured by AFM and n is the number of pixels in calculation. This process was repeated on four different samples with 5–10 macroscopically distant areas that were analysed on each sample after each step of the experiment. Histograms of the roughness distributions of the whole samples were made and fitted with two-term Gaussian. Areas of low and high roughness were calculated by integration of the Gaussian fit within the standard deviation range.

## Results

**Figure 2:**
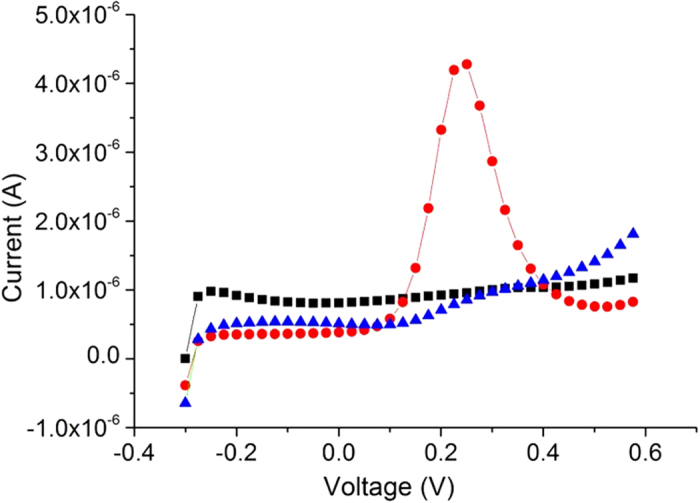
The electrochemical reversibility of the phosphorylation. Black (■) the HDGF 160–174 monolayer, red (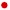) the monolayer after incubation with ERK2, blue (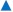) the monolayer after incubation with AP.

The process of phosphorylation and dephosphorylation was performed on gold macro electrodes and SWV measurements were taken after each step of the process (Fig. 2). The adsorbed HDGF 160–174 monolayer appears in the voltammogram as a nearly smooth line (peak current was 0.9 μA), indicating little oxidation of the redox species in the solution. This was caused by the peptide monolayer, which blocks the electron transfer to the electrode. After phosphorylation by ERK2, the oxidation peak current increased to 4.3 μA. This was caused by the addition of the phosphate groups to the peptides, which create disruption of the order in the monolayer. According to the proposed model, that has already been observed^25^, this disruption led to the formation of disordered areas or pinholes in the monolayer, which facilitate the electron transfer from the metal substrate to the solution. After dephosphorylation with AP the peak current decreased back to 0.9 μA. This also indicates that the phosphorylation process is reversible and harmless to the monolayer.

We characterized using AFM the roughness of HDGF 160–174 peptide monolayer on ultra-flat gold substrate after deposition, after phosphorylation and after dephosphorylation. These measurements are linking the macroscopic electrochemical observations to the microscopic interpretation. The roughness of the ultra-flat gold substrate was checked before the peptide deposition. The maximum peak-to-peak height in a 5 × 5 μm^2^ area was less than 2 nm and the RMS roughness was 0.18 ± 0.02 nm. Therefore, a flat substrate may be assumed for the examined 1 × 1 μm^2^ areas, with respect to the monolayer thickness and roughness.

**Figure 3:**
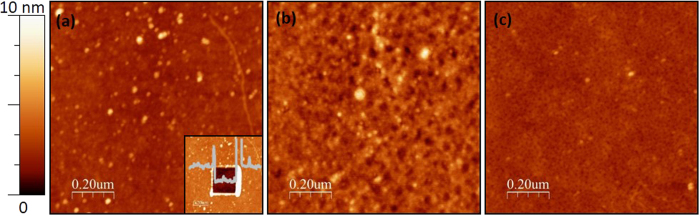
AFM topographic scan of peptide layer on ultra-flat gold after each step of the process. After peptide deposition; (**b**) After phosphorylation; (**c**) After dephosphorylation; Inset in (**a**) is the same area with a square scratch at the center. The profile on top demonstrates layer width (2.5 nm) and uniformity.

Figure 3 shows an example of AFM scans of 1 × 1 μm^2^ peptide monolayer areas after monolayer formation (Fig. 3a), after phosphorylation (Fig. 3b) and after dephosphorylation (Fig. 3c). All these scans were performed on the same gold surface for each sample.

As seen in Fig. 3a, the peptide monolayer is very smooth and uniform. The monolayer was scratched to evaluate its thickness (inset of Fig. 3a), which was found to be 2.5 nm. Another scratch was done in a smaller area inside the first scratch to eliminate the possibility of partial removal of the monolayer or damage to the substrate, which could affect the layer thickness measurements. After phosphorylation by ERK2 (Fig. 3b), the surface seems rougher, disordered and with “pinholes” in the peptide layer. Upon dephosphorylation (Fig. 3c) the “pinholes” shrink or disappear and the surface seems smooth again.

RMS roughness was measured and calculated at various 1 × 1 μm^2^ areas on the different samples at different parts of each sample, ~1 mm apart. Before phosphorylation the average RMS roughness was 0.47 ± 0.13 nm (8 scans of 1 × 1 μm^2^). After phosphorylation the roughness increased to 0.61 ± 0.17 nm (10 scans of 1 × 1 μm^2^) and after dephosphorylation it decreased to 0.50 ± 0.14 nm (11 scans of 1 × 1 μm^2^). These results show a small change in the average roughness, which we term “global roughness”, during the experiment. The error margins are smaller relative to the global roughness analysis method and therefore the scanned areas had close values. Thus, at the micron scale the surface is quite uniform.

Our results show visually and indicate numerically that phosphorylation of a peptide substrate by a kinase induces structural changes in the monolayer and increases the surface roughness while dephosphorylation reverses the process to its initial values. However, the observed changes in the average surface roughness in each stage are rather small and fall within each other’s margins of error.

To address this problem, extract finer information and get a deeper insight into the surface changes during the phosphorylation-dephosphorylation cycle, a higher resolution analysis was performed. Smaller areas of 100 × 100 nm^2^ were examined and a semi-automated process was implemented (see Methods for details). This enabled a much larger statistical base – up to 1000 areas at each step versus 5–14 areas previously^15^. We term the high resolution data analysis “local roughness” measurement (method schematics in Fig. 1).

**Figure 4:**
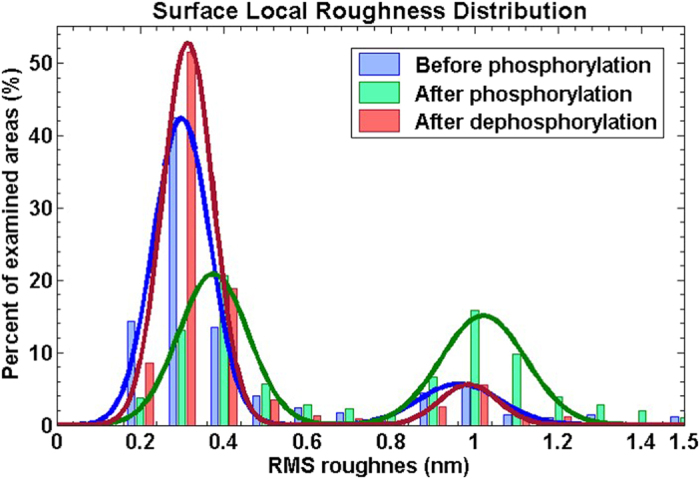
Surface local roughness distribution with a two-terms Gaussian fit. The blue and red lines, representing the distributions of the local roughness before phosphorylation and after dephosphorylation, respectively, are very close, and different from the local roughness after phosphorylation (green). The phosphorylated state distribution is upshifted to higher roughness values and contains many higher roughness domains.

Figure 4 presents the distribution of the local roughness at different stages of the phosphorylation–dephosphorylation cycle. Two distinct peaks appear in this distribution throughout the cycle, i.e. there are many areas with RMS roughness of 0.2–0.5 nm and many areas with RMS roughness of 0.9–1.2 nm, while very few areas have other roughness values.

Each distribution was fit to a two-terms Gaussian, represented by the solid lines in Fig. 4. Before phosphorylation the fit had mean values of 0.30 ± 0.10 nm and 0.96 ± 0.14 nm. After phosphorylation the mean values were 0.38 ± 0.12 nm and 1.02 ± 0.14 nm. After dephosphorylation the mean values were 0.31 ± 0.09 nm and 0.99 ± 0.10 nm. The small up-shift in the locations of the roughness peaks after phosphorylation, with respect to the peak positions of the deposited and dephosphorylated surfaces, is consistent with our earlier findings, as mentioned above^15^,^22^. Control experiments, in which the peptide layer was exposed to the reaction medium without the enzyme and then imaged by AFM and analysed similarly, showed no observable change in the layer roughness or in the peaks height ratio (Figure S1 in Supplementary information).

The local roughness analysis presented here shows that the peptide monolayers consist of low and high roughness domains, which could not be observed by the previously performed global analysis. Initially the peptide monolayer consists mostly of low roughness domains (blue line in Fig. 4). 73% of the scanned area has a low roughness of 0.31 ± 0.10 nm and only 14% has a higher roughness of 0.96 ± 0.14 nm (13% cover the rest of the roughness range of 0–20 nm, i.e. mostly contaminations on the surface). After phosphorylation, large part of the surface changes and becomes rougher (green line in Fig. 4) – 44% low roughness (0.38 ± 0.12 nm) and 40% high roughness (1.02 ± 0.14 nm). After dephosphorylation the ratio of low and high surface roughness areas was restored (red line in Fig. 4), 78% low (0.31 ± 0.09 nm) and 9% high roughness (0.99 ± 0.10 nm).

## Discussion

These results indicate that phosphorylation of the HDGF 160–174 peptide by ERK2 induces transition of domains in the peptides layer from ordered to disordered. Similarly, the dephosphorylation induces re-ordering of the layer. One may speculate that low roughness domains have dense packing while the high roughness domains are less ordered and contain defects and pinholes. At this point, however, there is no direct measurement of peptides packing in the monolayer at a molecular resolution.

We interpret the observed reversible roughness changes by two main contributions. One originates from a substantial amount of free space (i.e. pinholes) that is generated in the monolayer upon phosphorylation, thus damaging the insulating capacity of the peptides layer. This is supported by the morphological observation that the smooth HDGF 160–174 peptide monolayer behaves as a well packed self-assembled monolayer having dominant local roughness of ~0.3 nm (Fig. 4) and is pinhole free (Fig. 3a). Phosphorylation by ERK2 of this peptide layer leads to a pronounced increase in the RMS roughness to dominant local roughness of ~1.0 nm (Fig. 4) and to distinct pinholes formation (Fig. 3b). AP derived dephosphorylation restores the initial local roughness back to roughness of ~0.3 nm (Fig. 4) and the pinhole free smoother appearance of peptide monolayer (Fig. 3c). These structural transformations suggest reversible nanometric pinholes formation in the HDGF 160–174 peptide monolayer upon enzymatic phosphorylation-dephosphorylation reactions.

The second contribution is changes in the orientation and packing of peptides in the monolayer. The increased roughness upon phosphorylation could be related to the substitution of an alcohol group by a phosphate group in the monolayer. When the peptides in the monolayer are phosphorylated, two main parameters may contribute to order-disorder transition: a) steric effect of the bulky phosphate group containing larger hydration layer than that of the alcohol group, and b) the electrostatic repulsion between negatively charged neighbouring phosphates. Both parameters may disrupt the orientation and packing of the peptides and therefore the initial ordering of the native peptides monolayer. Although cis-trans isomerisation has been shown to take place in the XSPX phosphorylation motif^26^, and might cause to a change in the monolayer packing, it has already been proven by XPS^22^ and ATR-FTIR^27^ that a phosphate group is present upon enzymatic reaction, and more likely to cause the observed roughness change. Moreover, after dephosphorylation, the phosphate group is removed and is not observed in XPS and ATR-FTIR.

## Conclusions

In summary, a dense and uniform layer of the HDGF 160–174 peptide was formed on gold electrodes and ultra-flat gold surfaces. It was characterized by SWV and AFM before phosphorylation by ERK2, after phosphorylation and after dephosphorylation by AP. We observed a reversible electrochemical transformation of the peptide monolayer upon reversible phosphorylation and dephosphorylation and further confirmed our model of surface reaction by using a general local roughness analysis. While the experiment was performed on a very specific surface, the analysis method does not take into account any properties of the monolayer or the substrate. Therefore it can be applied to any kind of smooth surface to reveal additional information that is hidden in regular roughness analysis. While the average roughness of the peptides monolayer at 1 μm scale shows slight increase after phosphorylation, the changes are very small. Roughness calculations at 0.1 μm scale reveal a much more complicated picture – the surface is composed of two kinds of domains with different roughness. Phosphorylation changes the ratio between these two kinds and slightly increases the roughness of the smoother domains, presumably by introducing defects and disturbing the ordering of the densely packed domains. This process seems completely reversible as upon dephosphorylation the surface recovers to its initial morphology. The microscopic morphological analysis supports the model that provides a mechanistic insight into the behaviour of the monolayer in the electrochemical assays.

## Additional Information

**How to cite this article**: Zhuravel, R. *et al.* Atomic force microscopy characterization of kinase-mediated phosphorylation of a peptide monolayer. *Sci. Rep.* **6**, 36793; doi: 10.1038/srep36793 (2016).

**Publisher’s note:** Springer Nature remains neutral with regard to jurisdictional claims in published maps and institutional affiliations.

## Supplementary Material

Supplementary Information
