# Supplementary material for: Atomic force microscopy characterization of kinase-mediated phosphorylation of a peptide monolayer
Source: Sci Rep. 2016 Nov 14;6:36793. doi: 10.1038/srep36793 (PMC5107921; doi:10.1038/srep36793)
Supplement: Supplementary Information [file srep36793-s1.pdf]

Supplementary information

Atomic force microscopy characterization of kinase-mediated phosphorylation of a peptide monolayer

Roman Zhuravel[a], Einav Amit[a], Shir Elbaz[a], Dvir Rotem[a], Yu-Ju Chen[b], Assaf Friedler[a], Shlomo Yitzchaik[a]* and Danny Porath[a]*

[a] Institute of Chemistry and the Center for Nanoscience and Nanotechnology, the Hebrew University of Jerusalem, Safra Campus, Givat Ram, Jerusalem 91904, Israel.
[b] Institute of Chemistry, Academia Sinica, Taipei, Taiwan.

Control experiment – ERK2 kinase activity:

Sample preparation similar to the process described in the main manuscript (in the "methods" section):

Ultra-flat gold substrate was prepared by evaporation of 150 nm of gold on freshly cleaved mica, then attachment to 5X5 mm^2 glass using epoxy glue and removal of the mica by submerging in tetrahydrofuran (THF) for 3 minutes.

The monolayer was formed by submerging in solution of 0.1mM HDGF 160-174 in 100mM phosphate buffer. After AFM characterization the surface was submerged in solution of 80 mM Tris-HCl (pH = 7.5), 40 mM MgCl2, 0.2 mg/mL BSA and 100 µM ATP. This solution is similar to the solution of the phosphorylation step of the experiment, but without the kinase.

Results of the local roughness analysis presented bellow (figure S1) show no change in roughness distribution during the control experiment, indicating that ERK2 activity is indeed the reason for these changes in roughness.

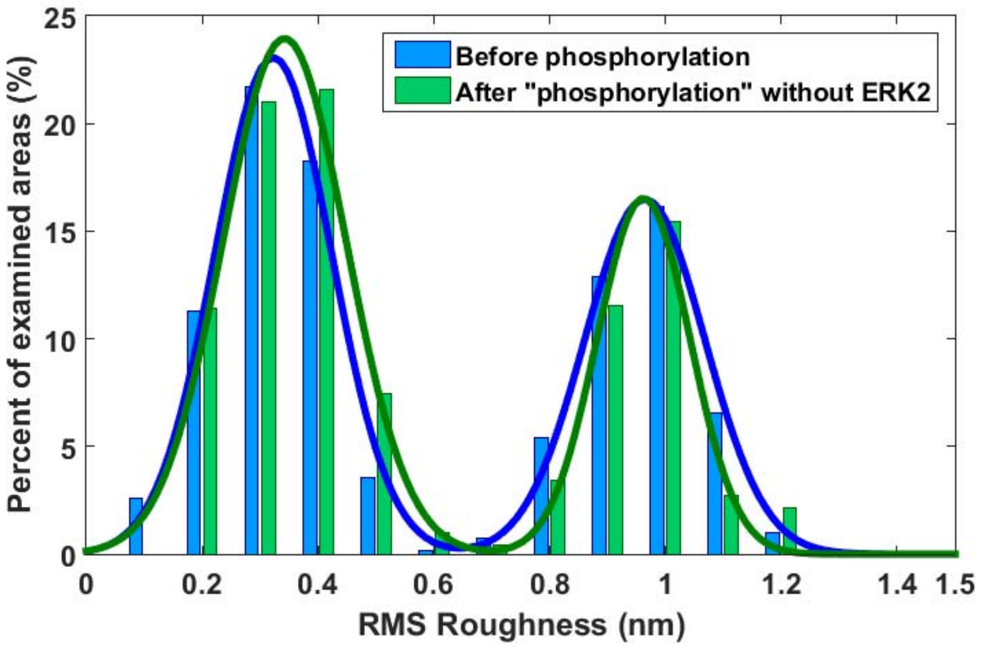

Figure S1 - Control measurement

Submerging the sample with the peptide monolayer into a solution that does not contain ERK2, did not effect the rpoughness distribution. The ratios between the two populations remain identical to those measured immidiately after formation of the monolayer.
